# Determination of Cefixime by a Validated Stability-Indicating HPLC Method and Identification of its Related Substances by LC-MS/MS Studies

**DOI:** 10.3797/scipharm.1301-15

**Published:** 2013-02-18

**Authors:** Zahra Talebpour, Hakimeh Pourabdollahi, Hasan Rafati, Asem Abdollahpour, Yusef Bashour, Hassan Y. Aboul-Enein

**Affiliations:** 1 Department of Chemistry, Faculty of Science, Alzahra University, Vanak, Tehran, Iran.; 2 Medicinal Plants & Drugs Research Institute, Shahid Beheshti University, G. C., Evin, Tehran, Iran.; 3 Jaber Pharmaceutical Companies, Tehran, Iran.; 4 Instrumental Analytical Chemistry Laboratory. Daana Pharma. Co. Factory: 15th Km, Tabriz-Tehran Rd, Tabriz, Iran.; 5 Department of Pharmaceutical and Medicinal Chemistry, National Research Centre, Dokki 12311 Cairo, Egypt.

**Keywords:** Cefixime, Related substances, LC-MS/MS, Bulk drug, Impurities

## Abstract

Cefixime is an important cephalosporin antibiotic that easily decomposes and releases different related substances in preparation and storage steps. The objective of the current study was to develop a simple, precise, and accurate isocratic liquid chromatography (LC) method for the determination of cefixime in the presence of its related substances generated from thermal stress in the bulk drug. The chromatographic conditions were comprised of a reversed-phase C18 column (4.6 × 250 mm, 5 μm) with a mobile phase composed of water: acetonitrile (85:15 v/v, with 0.5% formic acid) and ultraviolet detection (UV). Some thermal degradation products were identified using a proposed liquid chromatography-mass spectrometry method. Five peaks (A, B, C, D, and E impurities based on British Pharmacopoeia) were known and a few unknown peaks appeared in the thermal stress solution of cefixime. The linear regression analysis data for the calibration plot of the LC-UV method showed a good linear relationship in the concentration range 0.9–1000.0 μg mL^−1^. The recovery of the optimized method was between 94.6 and 98.4% and the inter- and intra-day relative standard deviations were less than 3.3%. The obtained results shown in the LC-UV proposed method can be conveniently used in a quality control laboratory for routine analysis of cefixime for the assay and related substances, as well as for the evaluation of stability samples of bulk drugs.

## Introduction

Cefixime, (6*R*,7*R*)-7-({(2*Z*)-2-(2-amino-1,3-thiazol-4-yl)-2-[(carboxymethoxy)imino]acetyl}-amino)-3-ethenyl-8-oxo-5-thia-1-azabicyclo[4.2.0]oct-2-ene-2-carboxylic acid, is an orally absorbed third generation cephalosporin antibiotic that was approved by the U.S. Food and Drug Administration in 1997 for the treatment of mild to moderate bacterial infections. It has a broad antibacterial spectrum against various Gram-positive and Gram-negative bacteria, including Haemophilus influenzae, Neisseria gonorrhoeae, Escherichia coli, and Klebsiella pneumoniae resistant to ampicillin, cephalexin, cefaclor, and trimethoprim-sulfamethoxazole. It is used for the treatment of susceptible infections, including gonorrhea, otitis media, pharyngitis, lower respiratory-tract infections such as bronchitis, and urinary-tract infections [1, 2].

The presence of impurities in drugs should be identified and quantified to evaluate efficiency and stability of the drug products as essential factors of their quality and safety [3–5]. A drug product, when not of a sufficient stability, can result in changes in physical characteristics (like hardness, dissolution rate, phase separation, etc.) as well as chemical characteristics (formation of high-risk decomposition substances) [6, 7]. Cefixime degrades under storage conditions (temperature and relative humidity) and its degradation products can cause undesirable side effects in patients [8, 9]. It is required that an analytical method be validated to demonstrate that impurities unique to cefixime do not interfere with, or that cefixime is separated from, its degradation products in the drug products.

Cefixime has been studied and determined by relatively few procedures such as spectrophotometric in dosage form [10, 11], fluorimetric in raw material and dosage forms [12], voltammetric in dosage forms and biological fluids [13, 14], high-performance liquid chromatographic in dosage forms and biological fluids [15–19], and high-performance thin-layer chromatographic in dosage form [20] methods. However, these methods are not specific for the determination of cefixime in the presence of its impurities and/or degradation products.

The aim of this study was to develop and validate a chromatographic method for the determination of cefixime in the presence of its related substances for the assessment of bulk drug purity and thermal stability of dosage forms. An isocratic liquid chromatographic (LC) method was performed with a C18 column at ambient temperature with an aqueous solution of water and acetonitrile using UV detection. Also, some cefixime-related substances have been identified using the spike of standard samples of known cefixime impurities and a liquid chromatography-tandem mass spectrometry (LC-MS/MS) method. The proposed LC-UV method has been validated based on the criteria of linearity, recovery, repeatability, and limits of detection (LOD) and quantification (LOQ).

## Results and Discussion

### Proposed LC-UV method

**Fig. 1:**
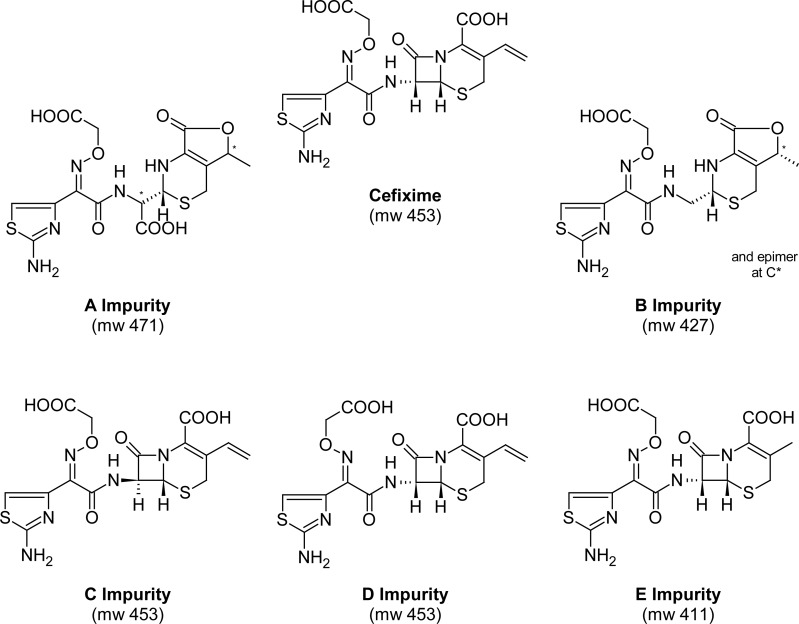
Chemical structures of cefixime and its proposed impurities in BP

Some impurities have been introduced for cefixime in BP. The structure and molecular weight of cefixime and its A–E impurities are shown in Fig. 1. Of them, impurity D is the main one and can be prepared in situ upon heating, but new decomposition compounds appear under this condition [21]. Based on the BP method for the analysis of related substances of cefixime, the C18 column was proposed at a temperature of 40°C using an aqueous solution of tetrabutylammonium hydroxide (pH=6.5 buffer phosphate): acetonitrile 75:25 v/v at a flow rate 1 mL min^−1^. The presence of the phosphate buffer caused this method tonot be compatible with the MS system, rendering the identification of the other cefixime-related substances impossible. Also, cefexime is a very sensitive compound, which easily decomposes under environmental conditions. It is beneficial if an MS system can qualitatively and quantitatively measure cefixime and its related compounds with high sensitivity and accuracy. The aim of this work suggests an LC-UV method that is compatible with an MS detector for on-line analyses of cefexime and related compounds.

In order to optimize the chromatographic conditions for the separation of cefixime from its related substances by the proposed LC-UV method, the cefixime thermal stress solution that contained some related substances was used. The analytical condition was selected after testing the different parameters such as protic and aprotic organic solvents, mobile phase composition, and flow rate to separate cefixime and its related substances.

The results showed that an increase in water content in the presence of acetonitrile, as an aprotic solvent, led to the increase in the resolution, but when the water content was ≥ 90%, the run time increased sharply without improvement in resolution. The best condition for peak capacity, resolution, and retention time was obtained using a mobile phase composed of water: acetonitrile (85:15 v/v) and 0.5% formic acid at a flow rate of 1.2 mL min^−1^. The presence of formic acid in the mobile phase resulted in repeatable and Gaussian peaks at ambient temperature. Under this optimum condition, the retention time of cefixime was 11.13 min and some additional peaks appeared in the chromatogram of the cefixime thermal stress solution.

### Identification of cefixime-related substances after thermal stress

In order to identify cefixime-related substances, two methods were performed. In the first method, available standard samples of some known impurities (A–E) based on the British Pharamcopeoia (BP), were spiked separately from the cefixime thermal stress solution. The obtained chromatograms, before and after spiking each impurity standard, were compared.

**Fig. 2:**
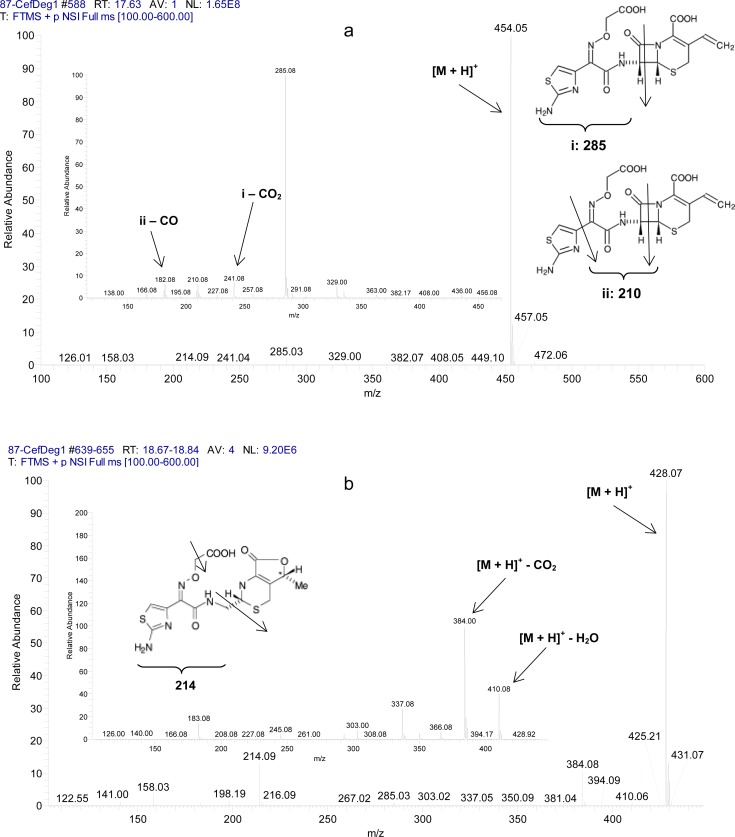
The full scan and MS/MS fragmentation of protonated molecular ions for a) cefixime and b) B impurity in the thermal stress solution.

Since the mobile phase of the proposed LC method was compatible with the MS system, LC-MS/MS was used as the second method for structure confirmation of cefixime-related substances in the thermal stress solution. The full mass scan and MS/MS spectra of the two main peaks are shown in Fig. 2. For the highest peak intensity, the m/z at 454 belonging to [M+H]^+^ of cefixime is shown. The MS/MS fragmentation of the cefixime-protonated molecular ion produced 285, 241, 210, and 182 m/z, which characterized the related chemical structures in Fig. 2a. For the peak with a relative retention time 1.2 to cefixime, the full mass spectrum indicated a protonated molecular ion at m/z 428 which can be related to impurity B. Moreover, the m/z at 214 in the full spectrum could be related to the chemical structure as shown in Fig. 2b and the m/z at 410 and 384 in the MS/MS fragmentation could be related to a loss of H_2_O and CO groups from the protonated molecular ion, respectively. Also, the protonated molecular ion peak of impurity A (472 m/z) was found for the peak at a relative retention time of 0.8 to cefixime.

The mass spectra of the peaks at relative retention times 1.3 and 1.7 to cefixime, showed molecular ions similar to cefixime. Being spiked with standards indicated that these peaks belong to impurities C and D, respectively.

**Fig. 3:**
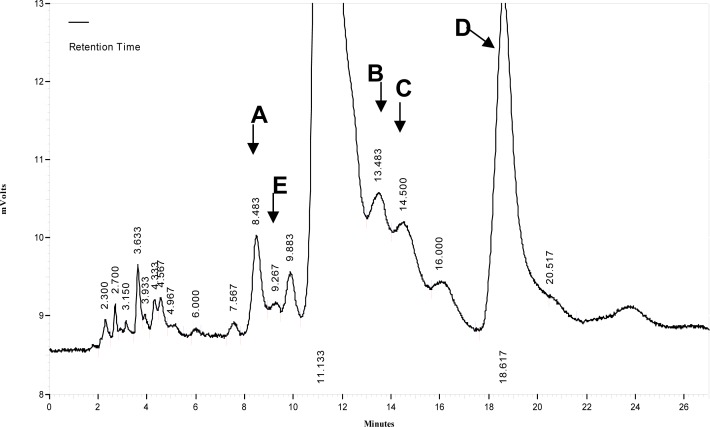
Characterized chromatogram of the cefixime thermal stress solution at 300 μg mL^−1^ using water: acetonitrile 85:15 v/v- 0.5% formic acid with flow rate 1.2 mL min^−1^. The structures of compounds A–E are shown in Fig. 1.

**Fig. 4:**
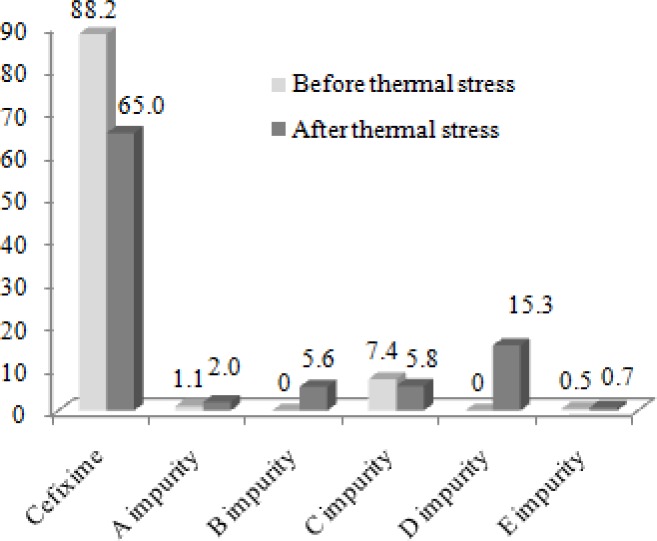
Cefixime and known related substance percentages based on relative peak area appeared in the chromatograms of fresh and thermal stress solutions of the bulk drug.

Based on the obtained results for the identification of cefixime-related substances using both methods, five peaks were identified as shown in Fig. 3. The percentages of cefixime and its known related substances in the bulk drug of the fresh and thermal stress solutions are summarized in Fig. 4. As can be seen, impurities D and B are not present in the fresh sample, however they were detected as the main degradation products in cefixime, when the temperature increased. Impurities A, C, and E had different intensities and were present in both the fresh and thermal stress samples of cefixime. Moreover, 2.8 % of the fresh and 5.6 % of the thermal stress solutions of cefixime in the bulk drug belonged to unknown compounds.

### Method validation

The system suitability was assessed by ten replicate analyses of cefixime at the concentration of 100 μg mL^−1^. Its retention time, capacity factor, and theoretical plates were obtained as 11.1 (±0.5%) min, 4.6, and 11035, respectively. The calibration plot of cefixime was obtained over the concentration range 0.1–1000 μg mL^−1^ using the proposed LC-UV method. The obtained linear regression equation and correlation coefficients were A = (6642 ± 45)C + (23 ± 12) and 0.9997, showing an excellent correlation between the peak area (A) and concentration of cefixime (C) over the range 0.9–1000.0 μg mL^−1^. The limit of detection and quantification, determined by the standard deviation method as described in the experimental section, were 0.26 and 0.90 μg mL^−1^, respectively, indicating that the method can be used for the detection and quantification of cefixime over a very wide concentration range.

The intra- and inter-day accuracy were calculated from the QC samples for cefixime at three concentration levels using two optimized mobile phases as described in the experimental section. The intra-day precision was evaluated by performing three determinations (n=3) at the same concentration, during the same day, under the same experimental conditions, and the inter-day precision was evaluated by comparing the assays on three different days. The obtained results for the intra- and inter-day accuracy and precision of cefixime using the proposed LC-UV method are summarized in Table 1. The results indicated good recoveries for the proposed assay (94.6–98.4%) with the relative standard deviation less than 3.3%.

### Analysis of the pharmaceutical products

The validated method described above was used to determine the total drug content of two commercially available brands of cefixime tablets containing 200 and 400 mg cefixime. The recoveries of cefixime from 200 and 400 mg tablets were 97.1% (RSD: 0.4%) and 94.5% (RSD: 1.1%), respectively. The results of the assay indicated that the method is selective for the analysis of cefixime without interference from its related substances and the tablet excipients.

## Experimental

### Chemicals

Standard materials of cefixime trihydrate and its impurities A–E were obtained from Hanmi (Seoul, Korea). Methanol and acetonitrile of HPLC grade were purchased from Caledon (Ontario, Canada). The water used for the preparation of the mobile phase and solutions was obtained through the Milli-Q water purification system. Other chemicals used at analytical reagent grade were purchased from Merck (Darmstadt, Germany).

### Instruments

An HPLC system containing a Kontron Model 420 pump, valve injector Rheodyne (Rohnert Park, CA, USA) equipped with a 20 μL loop and a Kontron Model 432 detector set to 254 nm, was used with Chromgate software (Knauer, Berlin, Germany) as the data acquisition system. The separation was performed on a C18 column (4.6 × 250 mm × 5 μm, Knauer, Berlin, Germany). The mobile phase was used in isocratic mode with a flow rate in the range of 0.8–1.2 mL min^−1^. The composition of the mobile phase for the LC-UV method varied in the optimization process from 80:20 to 90:10 v/v of water: acetonitrile including 0.5% formic acid.

A ThermoFisher Scientific (Bremen, Germany) ion trap mass spectrometer (model LCQ, mass range m/z 10–2000) equipped with an ESI interface was used in the present study. Instrument control, data acquisition, and processing were conducted by the Xcalibur software. Typical positive ESI-MS conditions were: capillary voltage 3.0 kV and skimmer cone voltage −20 V. The collision energy for MS/MS was in the range of 25–40 eV. The mobile phase was composed of water: acetonitrile (85:15 v/v) including 0.5% formic acid.

### Preparation of standard solutions

A fresh solution of the pure cefixime trihydrate at 300 μg mL^−1^ concentration level was prepared by dissolving appropriate amounts of it in the mobile phase. Based on the British Pharmacopoeia (BP) method [21], the thermal stress sample of cefixime was studied by subjecting the cefixime standard solution to paraffin oil heated up to 80°C for 1 h. This procedure was repeated for the bulk drug of cefixime.

### Preparation of the pharmaceutical product solutions

Ten tablets of two dosage forms of cefixime (containing 200 and 400 mg cefixime) were accurately weighed and finely powdered by mortar and pestle. An adequate amount of this powder, corresponding to a solution at a concentration level of 350 μg mL^−1^, was weighed, transferred into a 100 mL calibrated flask, and completed to the volume with the mobile phase. The contents of the flask were sonicated for 15 min to affect complete dissolution. The resulting solution was filtered using a 0.45 μm filter into standard analytical glass vials and 20 μL were injected into the HPLC to verify the label claim pertaining to cefixime. Each analysis was replicated three times. The drug content in a tablet was determined by referring to the regression equations.

### Method validation

A calibration curve was prepared using the proposed LC-UV method with eight calibrators over a concentration range of 0.1–1000 μg mL^−1^ for cefixime and constructed by plotting peak areas against the respective concentrations. The linearity was evaluated by the least-squares regression method, which was used to calculate the r-value, y-intercept, and slope of the regression line.

The limits of detection (LOD) and quantization (LOQ) were calculated by the method based on the standard deviation (σ) of the blank and the slope (S) of the calibration plot, using the formulae LOD = 3.3σ/S and LOQ =10σ/S.

The accuracy and precision of the method were determined for the drug substance by analyzing the standard quality control (QC) samples at three concentrations of cefixime (50, 350, and 650 μg mL^−1^) using the proposed LC-UV method. Accuracy was established by evaluating the determined amount from the QC samples and comparing that to the respective nominal value expressed as percent recovery. The method precision was established by injecting three replicate standard QC samples at each concentration level for the intra-day precision and on three days for the inter-day precision. Precision was expressed by the relative standard deviation (RSD, %) of the recoveries.

## Figures and Tables

**Tab. 1.** Accuracy and precision of the proposed LC-UV method for the determination of cefixime in the presence of its related substances

**Precision (RSD %) n=3**	**Accuracy (Recovery %) n=3**	**Inter-day**		**Intra-day**	

		**Precision (RSD^a^ %) n=3**	**Accuracy (Recovery %) n=3**	**Found Concentr. (μg mL^−1^)**	**Added Concentr. (μg mL^−1^)**
3.3	94.6	0.8	98.1	49.1	50
0.6	95.7	0.3	96.3	337.0	350
0.7	98.4	0.03	98.0	636.7	650

a Relative standard deviation.
